# Bridging the Gap between RF and Optical Patch Antenna Analysis via the Cavity Model

**DOI:** 10.1038/srep15941

**Published:** 2015-11-02

**Authors:** G. S. Unal, M. I. Aksun

**Affiliations:** 1 Electrical and Electronics Engineering, Koç University, Istanbul, Turkey

## Abstract

Although optical antennas with a variety of shapes and for a variety of applications have been proposed and studied, they are still in their infancy compared to their radio frequency (rf) counterparts. Optical antennas have mainly utilized the geometrical attributes of rf antennas rather than the analysis tools that have been the source of intuition for antenna engineers in rf. This study intends to narrow the gap of experience and intuition in the design of optical patch antennas by introducing an easy-to-understand and easy-to-implement analysis tool in rf, namely, the cavity model, into the optical regime. The importance of this approach is not only its simplicity in understanding and implementation but also its applicability to a broad class of patch antennas and, more importantly, its ability to provide the intuition needed to predict the outcome without going through the trial-and-error simulations with no or little intuitive guidance by the user.

A wealth of knowledge and experience in the design and analysis of antennas is available in the radio frequency (rf) regime, with a great potential for inspiration and assistance in the optical regime. However, only recently have some uses of antennas entered into man-made optics^1^,^2^,^3^,^4^. Their late arrival in optical systems is mainly due to the lack of fabrication techniques with nanometer precision. Since the fabrication techniques were improved and became accessible to researchers, there has been a flurry of activities and interests in optical antennas and their applications^5^,^6^,^7^,^8^,^9^,^10^,^11^. However, optical antennas have mainly exploited the geometric shapes of rf antennas (e.g., dipole, bow-tie and yagi-uda) and some of their fundamental performance characteristics, such as the radiation pattern, directivity and gain, and input impedance^10^,^11^, even though there are a few well-developed, tested and successfully utilized analysis tools in rf that may provide intuition for the operation and design of optical antennas. Inspired by this assessment, in this study, we have focused on transferring the accumulated knowledge in one of the most widely employed and versatile antenna configurations in rf, the patch antennas, to the optical frequencies, with special emphasis given to their modeling and the computational aspect. As such, this study intends to open a new venue of new design approaches for various functionalities of optical patch antennas.

Patch antennas, since their ideation in the 1950s^12^ and realization in the 1970s^13^, have been one of the most studied and innovative classes of antennas in rf, with several variations in patch shape, feeding and substrate configurations, analytical and semi-analytical models and design techniques^14^. However, only recently have there been a few applications of the patch antennas in the optical frequencies to achieve large collection efficiency, better sensing and better control of the radiation pattern^15^,^16^,^17^,^18^,^19^. Although some of the advantages of the rf patch antennas may not directly translate to the optical frequencies, at least for now, other advances, such as polarization diversity, dual or multiple functionalities and variety in design, may become useful and play important roles in optics. For the characterization of these antennas in optics, either a simple transmission line model with little intuition and limited or no applicability outside the rectangular patches^15^,^16^, or a full-wave approach with good accuracy but no intuition^17^,^18^,^19^ has been employed. However, consideration of a metal patch as a surface plasmon cavity^17^ has been an inspiration for this study to employ the cavity model in the analysis and design of the optical patch antennas.

## Cavity Model

**Figure 1:**
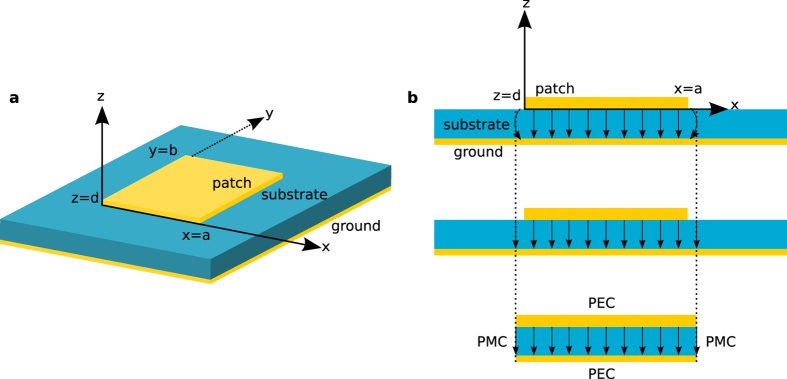
A typical rectangular patch antenna in rf and its cavity equivalent. (**a**) A metallic patch with dimensions *a* × *b* and thickness *d* on a dielectric substrate backed by a metal ground plane. (**b**) A cross-sectional view of the equivalent cavity of the rf patch antenna. The equivalent cavity is formed by perfect electrical conductors (PEC) and perfect magnetic conductors (PMC) and has dimensions slightly larger than the actual patch to account for the fringing fields. The radiation from the rf patch antenna can be interpreted either from the patch or its cavity equivalent, due to the equivalence theorem.

A typical configuration of the patch antennas in rf consists of a piece of metal trace on a substrate backed by a metal ground plane, as shown in Fig. 1a for a rectangular patch shape.

The basic operation of rf patch antennas can be summarized in a few sentences, with reference to Fig. 1b, to provide context for the following discussions. Regardless of the operating mode, transmitting or receiving, the current on the patch and the associated fields between the patch and the ground plane are excited either by a feed or incident wave. Because the thickness of the substrate is usually a fraction of the wavelength of operation, the electric field components parallel to the metal planes are negligible between the planes, yielding the *E*_*z*_(*x*, *y*), *H*_*x*_(*x*, *y*) and *H*_*y*_(*x*, *y*) field components only. If the characteristic length of the patch (*a* or *b* for the rectangular patch) is close to *λ*/2 or its integer multiple, the patch element resonates and sustains relatively large currents and associated fields in the structure, becoming the source of radiation. Due to the equivalence theorem, there are two interpretations of the radiation mechanism of such antennas^20^: the antenna can be viewed either (i) as the patch with the resonating current on, or equivalently, (ii) as a cavity formed by the patch and the ground plane enclosed laterally by the slot-type radiators with the resonating field inside (Fig. 1b). Consequently, the cavity interpretation inspired the cavity model, which has proven to be very efficient, intuitive, and easy to understand and use for the analysis and design of the patch antennas of some canonical shapes^21^,^22^,^23^. The salient feature of the approach is that it can provide analytical expressions for the fields inside the cavity where the closed-form solutions Ψ_*mn*_ of the scalar wave equation ∇^2^Ψ + *k*^2^Ψ = −*δ* are possible with the appropriate boundary conditions. Once the equivalence is established between the cavity and the patch, the fields inside the cavity can be expressed as a superposition of all possible orthogonal modes of the cavity as


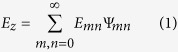


where 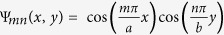 for the rectangular patch. Therefore, all relevant antenna parameters, such as the current distribution on the patch and radiation pattern, can be obtained in closed forms, enabling the computation of these parameters with ease and the design of an antenna with great intuition.

Despite the fact that the cavity model seemingly requires a physical cavity, as in the Metal-Insulator-Metal (MIM) configuration shown in Fig. 1b, we have realized that even a free-standing metal patch, i.e., the Insulator-Metal-Insulator (IMI) configuration, of one of the canonical shapes^22^ can be considered as a patch antenna suitable for the cavity model in optics, provided that the reflection coefficients from the edges of the patch can be obtained. The key idea behind the use of the cavity model in optics for such configurations is that the surface plasmon polariton (SPP) modes supported by the patch are equivalent to and agree well with the modes predicted by the cavity model because the radiation from the side walls of the cavity is equivalent to the radiation from the surface current on the patch^20^. Therefore, knowing the current distribution of a mode or the combination of the modes of an equivalent cavity guides us where to locate the source on the patch to excite that mode and provides intuition on how to tune the resonance, radiation pattern and polarization of the antenna, as discussed below.

## Theory

From the basic principles, the use of the cavity model for the optical patch antennas is possible as long as the differences of the wave-matter interactions in these frequency regimes, and in turn the resonance conditions, are properly addressed. The main difference relevant to the subject matter of this study is the wave-matter interaction at the interface between the dielectric and metal, which manifests itself as the surface current in rf and the SPPs in the optical frequencies. Whereas the SPPs are highly localized to the dielectric-metal interface and propagate along the interface with a wavelength smaller than the wavelength of incidence, the electromagnetic fields due to the surface currents in rf extend to the surrounding region with the wavelength of incidence. Therefore, for antennas operating at the optical wavelengths, the resonance of the structure is fundamentally defined by the SPPs and their interactions with the antenna structure.

It is a straightforward matter to obtain the dispersion relations for the SPPs analytically in a symmetric three-layer system^24^ and numerically in multilayered structures via the poles of the reflection or generalized reflection coefficients^25^. Because the MIM configuration in optics is the natural extension of the cavity in rf, such structures were the first to implement the cavity model with success (see the Supplementary Fig. S5). However, in this study, the implementation of the cavity model is demonstrated on a non-intuitive structure, that is, an IMI configuration using a homogenous environment (e.g., air) as insulator. Hence, to begin with, the propagation constant for the SPP along the metal patch with a thickness *d* in a homogenous environment is obtained from


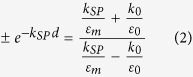


where *ε*_*m*_ represents the dielectric function of the metal and *k*_*SP*_ = *k*_0_*n*_*SP*_ + *iα*_*SP*_/2. Then, the resonant length of the patch antenna *l* for the fundamental mode can be obtained from the resonance condition of the SPP that undergoes reflections from the edges of the patch, as


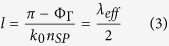


where Φ_Γ_ is the phase of the reflection coefficient Γ defined at the edges of the patch and is of significant importance to accurately define the resonant length of the patch. Based on the previous work^26^, the reflection coefficient defined at the edges of a finite IMI configuration can be obtained from


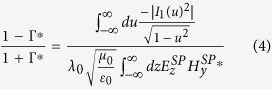


where 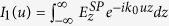, and 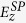 and 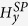 are the SPP fields supported by the structure. Once the effective wavelength, and in turn the resonant length of the antenna, has been determined, *k*_*eff*_ can be calculated as


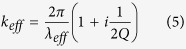


where the quality factor 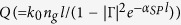 is obtained by the approximate Fabry-Perot model that accounts for the absorption and radiation losses^27^, and *n*_*g*_ (= *n*_*SP*_ − *λ*∂*n*_*SP*_/∂*λ*) is the group index of the IMI configuration.

With the relevant parameters of the geometry defined in optics, one can use the expression of the current distribution on the patch obtained by the cavity model to decide where to feed the patch to excite a specific mode, and subsequently, to obtain the required radiation and polarization patterns.

## Results

Although several patch antennas in MIM and IMI configurations were studied, and a few typical results are provided in the Supplementary Information to illustrate the details of the method, we have chosen a free-standing, rectangular gold patch with thickness *d* = 50 nm, as illuminated by a dipole source (*λ*_0_ = 1100 nm) to excite the SPP at the gold-air interface, with *k*_*SP*_( = 5.8 × 10^6^ + *i*1.05 × 10^4^ rad/m) (*λ*_*SP*_ = 1083 nm) from equation (2). It is safe to assume that *k*_*SP*_ is independent of the length and width of the antenna because the resonator (i.e., the patch) is not well below the diffraction limit. Therefore, the effective wavelength is computed as *λ*_*eff*_ = 540 nm from equation (3) by using the reflection coefficient data 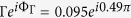 obtained from equation (4).

**Figure 2:**
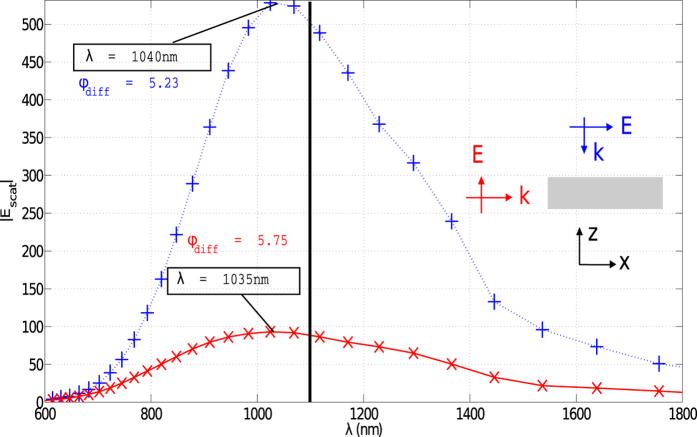
Magnitude of the scattered electric field from a square patch antenna due to a plane wave excitation with TM (blue pluses) and TE (red crosses) polarizations, as depicted in the inset. Parameters of the patch: *a* = *b* = 270 nm, *d* = 50 nm, and the patch is made out of gold. The black vertical line shows the resonant wavelength of *λ*_*res*_ = 1100 nm.

To verify the computed data for the effective wavelength and the reflection coefficient, the total scattered field from the gold patch antenna, with the dimensions of *a* = *b* = *λ*_*eff*_ /2 = 270 nm and thickness *d* = 50 nm, as a function of the wavelength, was obtained from a commercial-grade simulator based on the finite-difference time-domain (FDTD) method^28^ (Fig. 2). Knowing that the scattered field intensity is at its maximum at resonance, the peak of the scattered field upon a plane wave excitation in Fig. 2 shows the resonant wavelength at approximately 1040 nm, which is off by 60 nm, corresponding to a 5° difference in the phase of the reflection coefficient and a 1.5 nm difference in the antenna resonant length. Considering that the fabrication precision is on the order of a few nanometers, the calculated effective wavelength, based on the analytical calculation of the reflection coefficient in equation (4), can be used for all practical purposes. As a result, the quality factor *Q* and the effective wavenumber *k*_*eff*_ were obtained from equation (5) and the following inline expression of *Q* as (1.16 × 10^7^ + *i*3.52 × 10^6^ rad/m) and 1.65, respectively.

**Figure 3:**
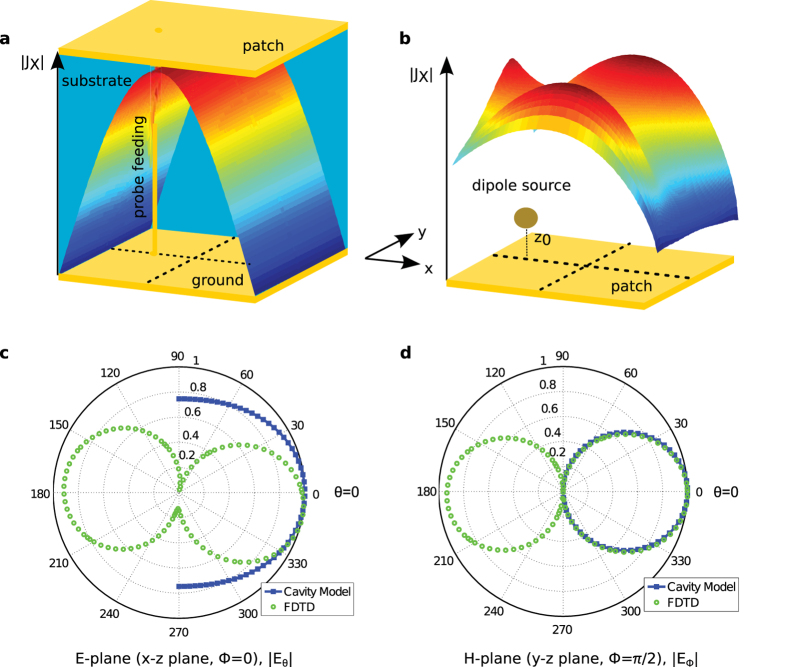
A square gold patch antenna (*a* = *b* = 270 nm, *d* = 50 nm, *x*_0_ = *a*/4, *y*_0_ = *b*/2) with the cavity model implementation: the current distribution and the radiation pattern for the *TM*_10_ mode. (**a**) The magnitude of the current density |*J*_*x*_| calculated by the cavity model when fed by a probe feed. (**b**) The magnitude of the current density |*J*_*x*_| calculated by the Maxwell solver when fed by a dipole at *z*_0_ = −15 nm. (**c**,**d**) The radiation patterns: |*E*_*θ*_| in the E-plane (*x* − *z*, *ϕ* = 0°) and |*E*_*ϕ*_| in the H-plane (*y* − *z*, *ϕ* = 90°). Circles (green) represent the scattered field data from the FDTD method, whereas the squares (blue) are from the cavity model. Only the upper halves of the patterns are given for the cavity model because of the PEC ground plane approximation.

Once the relevant data for the application of the cavity model have been verified, it becomes a straightforward implementation of the cavity model to define the modes of the antenna in terms of the modes of the cavity, as given in equation (1), which are referred to as *TM*_*mn*_ modes. The field profile corresponding to a specific mode dictates where to position the source, whether it is a nanoparticle (metallic or molecular) above/below the patch or a small discontinuity (e.g., gap or dent) on the patch, to excite the required current distribution on the patch and, in turn, the radiation pattern and the polarization of the radiation. In other words, one needs to position the source, with the dipole moment *μ*, where it couples the most to the electric field of the desired mode and to less or none of the undesired mode because the change of the energy dissipation of a dipole in an inhomogeneous environment is proportional to Im{μ^*^ ⋅ *E*_*s*_}^29^. Based on the modal profile in equation (1) predicted by the cavity model, |*E*_*z*_| is zero (has a null) at *y* = *b*/2 for the *TM*_01_ mode and at *x* = *a*/2 for the *TM*_10_ mode. Therefore, as an example, positioning an electrical dipole feed at (*x*_0_ ≠ *a*/2, *y*_0_ = *b*/2, *z*_0_ = −15 nm) will excite only the *TM*_10_ mode, as shown in Fig. 3a,b by the current distribution on the patch obtained by the cavity model and the Maxwell solver. To further emphasize and verify the concept, the current distribution on the patch due to a source in the middle, i.e., at (*x*_0_ = *a*/2, *y*_0_ = *b*/2), for which neither mode can be excited theoretically, was provided in comparison with the current distribution for the *TM*_10_ mode in the Supplementary Fig. S3. Although there is no actual cavity for the optical case in Fig. 3b, the current distributions in Fig. 3a,b and the radiation patterns in Fig. 3c,d show that the predictions of the cavity model are in reasonable agreement with the actual results and can provide a good starting point in the design of an optical patch antenna.

After having extensively studied the current distributions and the radiation patterns in the *E*- and *H*-planes for the *TM*_10_ and *TM*_01_ modes for various feed locations, here are some of the observations: (i) the radiation patterns in the *H*-plane for the optical patch antennas agree well with the cavity model predictions (Fig. 3d); (ii) the current density along the resonating length shows a standing wave nature, but it is not as symmetric as it is for the cavity model, which can be attributed to the combination of the radiation loss, metallic loss and the asymmetry in the location of the feed from the edges (Fig. 3c,d); (iii) the radiation pattern in the *E*-plane is more directive and tilted slightly from the broadside, as would be expected from a single metal antenna with slightly asymmetric current distribution (Fig. 3c), and (iv) the symmetric feed locations with respect to the center of the patch provide mirror image symmetric profiles both for the current density and the radiation pattern (see the Supplementary Figs S1 and S2), which may play an important role in sensing applications due to the ability to differentiate between the different feed locations that generate the same antenna mode, a behavior that could not be observed in rf.

**Figure 4:**
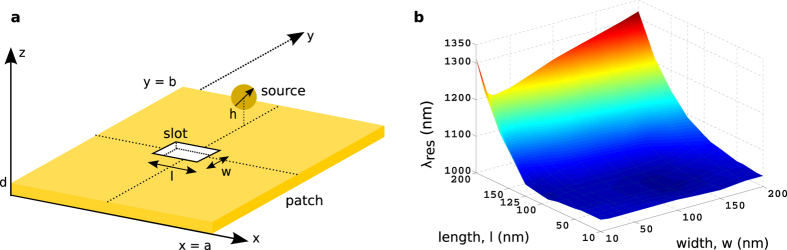
Tuning the resonant wavelength of an optical patch antenna. (**a**) Optical patch antenna with a slot of length *l* and width *w*. Other parameters are as follows: *a* = *b* = 270 nm, *d* = 50 nm and *h* = *z*_0_ = −15 nm. (**b**) Resonant wavelength (*λ*_*res*_) of the patch antenna obtained by the FDTD method as a function of the length *l* and width *w* of the slot.

Based on the study of the current distributions on and the radiation patterns from the rectangular patches, it has been established that the optical patch antennas can be designed using the cavity model, at least for the initial design phase, where one can define the dimensions of the patch and the feed location(s) according to the intended frequency of operation, the radiation pattern and the polarization of the radiation. Having gained intuition for the current distribution on the patch by the cavity model, one can introduce and translate some of the tools that are available for the rf patch antennas into optics. For example, tuning the resonant frequency of a mode can be achieved by introducing a thin slot on the patch that effectively increases the resonant length of that mode, and in turn, decreases the resonant frequency, as shown in Fig. 4a for the implementation in optics. Its circuit interpretation is simple because the narrow slot can introduce a parallel capacitor *C*_*slot*_(∝ *ld*/*w*) to the equivalent resonant circuit of the mode (*m*,*n*)^23^, resulting in a shift of the resonant frequency defined by 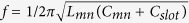 (Fig. 4b). Although the frequency tuning by introducing a slot on the patch and its simple circuit interpretation have been successfully demonstrated in the optical regime, due to the complex behavior of the resonant wavelength, as evidenced in Fig. 4b, a slot of different widths and lengths in a patch needs to be studied further.

**Figure 5:**
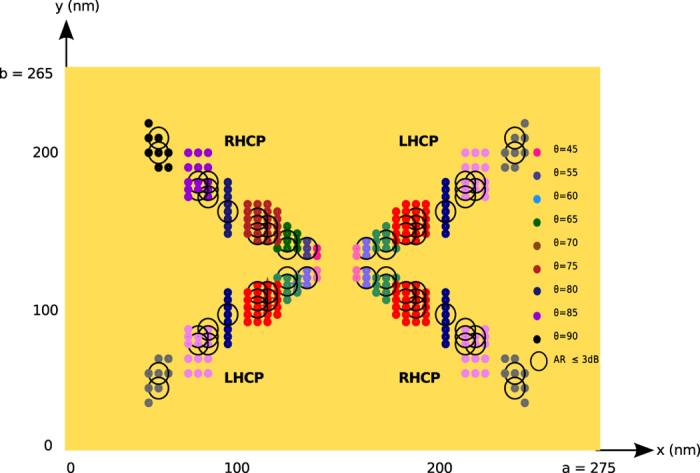
The locations (dots) of the dipole source for the CP operation of an optical patch antenna for a set of polarizations (colors of the dots) of the source, defined by the polar angle on the *x* − *z* plane. The antenna is made out of gold with the following parameters: *a* = 275 nm, *b* = 265 nm, *d* = 50 nm and *z*_0_ = −15 nm. The data in the second quadrant (marked by the dark colors) were obtained by the Maxwell solver, whereas the rest (marked by the lighter colors) were deduced from the symmetry of the geometry. The dots represent the locations of the feed for the CP operation with *AR* ≤ 6 dB and the circles are for *AR* ≤ 3 dB.

Another attractive feature of the rf patch antennas is their ability to produce and control the polarization of the radiation by simply selecting the position of the feed, which may be useful in sensing and imaging applications in optics. For the rf patch antennas, a simple cavity model-based approach is used to find the locations of the feed to achieve circularly polarized (CP) radiation^23^. The model uses the radiation fields at the broadside of the antenna, due to the magnetic current densities for the *TM*_01_ and *TM*_10_ modes at the edges of the cavity (on the PMC walls in Fig. 1b) to enforce the condition for the CP operation, that is, 
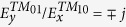
 in the far field. However, because the current distributions obtained by the cavity model for the *TM*_01_ and *TM*_10_ modes on the antenna do not exactly match the actual current distributions, as shown in Fig. 3a,b for the *TM*_10_ mode, especially near the edges of the patch, the phases of the electric field components in the far field are expected to show some deviations from those obtained by the cavity model, whereas the magnitudes of the fields are stationary in the far-field zone with respect to small deviations in the current distribution. As would have been inferred by the preceding assessment, the direct application of the cavity model-based approach to the optical patch antenna discussed and verified earlier (Fig. 1b) was not able to yield the positions of the vertical dipole on the patch to generate the CP operation. However, for optical patch antennas, there is one more degree of freedom for the parameters of the feed to choose, that is, the polarization of the emitter, in addition to its projected location on the patch. Hence, using the polarization of the source as an additional parameter, the loci of the feed for the right-handed and left-handed CP operations are obtained from the Maxwell solver for the optical rectangular patch antenna, as shown in Fig. 5. As a conclusion, the CP operation of a free-standing patch antenna is possible for a dipole source whose locations have to be carefully selected for a given polarization of the source, or vice versa. This feature is an important difference from the rf operation because it may lead to a better sensing of the polarizations of nano-emitters, such as fluorescent molecules.

**Figure 6:**
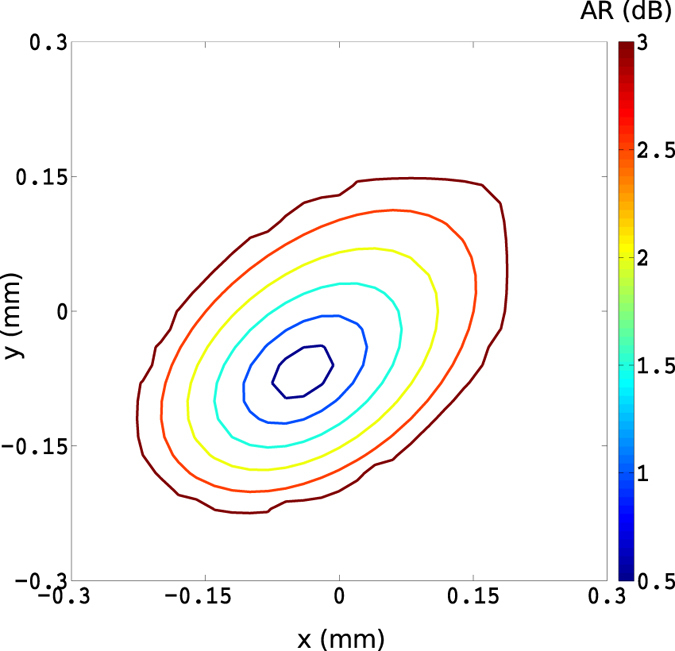
The contour plot of the axial ratio in dB. The antenna is excited by a dipole source at (*x*_0_ = 72.5 nm, *y*_0_ = 182.5 nm, *z*_0_ = −15 nm) with the polarization angle of *θ* = 85° on the *x* − *z* plane. The dimensions of the gold rectangular antenna are as follows: *a* = 275 nm, *b* = 265 nm, *d* = 50 nm.

To assess the quality of the CP operation, the contour plots of the axial ratio (*AR*) are given in Fig. 6, where the contours of *AR* ≤ 3 dB are shown at the *z* = *const*. plane in the far-field. Almost perfect CP operation, with *AR* = 0.5 dB, is achieved by the antenna when it is fed by a dipole emitter at (*x*_0_ = 72.5 nm, *y*_0_ = 182.5 nm, *z*_0_ = −15 nm) with the polarization angle of *θ* = 85° on the *x* − *z* plane.

## Conclusion

In this study, the main focus was the transfer of the established knowledge and practices of rf patch antennas into optics, with special emphasis given to the cavity model that not only provides simple and analytical solutions with reasonable accuracy but also helps to analyze and design patch antennas with an understanding of the operating mechanism and physical phenomena. Because the cavity model was originally proposed and developed for rf patch antennas, which are surrounded by perfect electrical and magnetic conductors, its mapping into optics, where a patch antenna may not geometrically appear as a cavity and the metals employed are no longer perfect conductors, has been developed and tested. The cavity model equivalent in optics has achieved the intended goal of providing intuition on the operation of optical patch antennas. As a result, the current distribution on an optical patch antenna and the associated radiation patterns can be predicted for a given location of the feed. Therefore, one can intuitively play with the patch geometry, as well as the other parameters of the patch, to match the radiation response to the requirements and specifications of the problem at hand. In addition, some of the tools that have been used for rf patch antennas, such as adding a slot to tune the resonance and designing the CP operation, have been introduced and demonstrated in optics. As a final note, the same analysis and intuitions can now be carried over to a range of patch geometries where the cavity model can be used.

## Additional Information

**How to cite this article**: Unal, G. S. and Aksun, M. I. Bridging the Gap between RF and Optical Patch Antenna Analysis via the Cavity Model. *Sci. Rep.* **5**, 15941; doi: 10.1038/srep15941 (2015).

## Supplementary Material

Supplementary Information
