# Supplementary material for: Bridging the Gap between RF and Optical Patch Antenna Analysis via the Cavity Model
Source: Sci Rep. 2015 Nov 2;5:15941. doi: 10.1038/srep15941 (PMC4629117; doi:10.1038/srep15941)
Supplement: Supplementary Information [file srep15941-s1.pdf]

Bridging the Gap between RF and Optical Patch Antenna Analysis via the Cavity Model

G. S. Unal[1] and M. I. Aksun[1,*]

[1]Electrical and Electronics Engineering, Koç University, Istanbul, Turkey.
[*]iaksun@ku.edu.tr

Supplementary Information

In the main text, the idea of introducing the cavity model for the analysis and design of the optical patch antennas was demonstrated and verified for a free-standing patch antenna, i.e., a square gold patch excited by a dipole ($\lambda_0 = 1100$ nm) in an IMI configuration. For the sake of brevity, the breadth of the study was not included in the main text. It is the aim of this Supplementary Information to provide more examples to further verify the validity of the proposed method for various wavelengths, sizes and materials.

In this regard, the current distribution and the radiation patterns for the TM_{10} mode, when excited by a dipole at ($x_0 = 3a/4, y_0 = b/2$), are given in Fig. 1 for the same antenna as in the main text, i.e., the square gold patch with $a = b = 270$ nm, $d = 50$ nm and $\lambda_0 = 1100$ nm. The symmetric feed locations with respect to the center of the patch, ($x_0 = a/4, y_0 = b/2$) and ($x_0 = 3a/4, y_0 = b/2$), result in the symmetric current distributions with respect to the center and the symmetric tilt in the radiation patterns in the E-plane with respect to the broad side of the antenna, $\theta = 0^o$. However, for patch antennas in rf, we expect to observe the same current distributions and radiation patterns due to the symmetric feed positions with respect to the center because they would excite the same mode profile in the cavity. To further emphasize the difference in the radiation patterns corresponding to the symmetric feed locations because of their potential use in optics, the radiation patterns in the E-plane are given comparatively in Fig. 2, where the tilts in the direction of the main beam are clearly observed.

The cavity model predicts zero coupling to the TM_{10} and TM_{01} modes if the source is placed in the middle, ($x_0 = a/2, y_0 = b/2$), resulting in a negligible current distribution on the patch. To verify this deduction of the cavity model in the same optical patch antenna, the corresponding current distribution on the patch is provided in Fig. 3 in comparison with the current distributions for the TM_{10} mode fed by a dipole located at ($x_0 = a/4, y_0 = b/2$).

Because we have only presented the data for the TM_{10} mode in all the examples, the same patch antenna was also studied for the TM_{01} mode, with a dipole feed at ($x_0 = a/2, y_0 = b/4$), for which the current distribution and the radiation patterns are shown in Fig. 4.

Because the natural extension of an rf patch antenna is a patch in an MIM configuration in optics, a new square gold patch antenna in the MIM configuration ($a = b = 366$ nm, $d_{metal} = d_{air} = 50$ nm) was studied, with all the verifications discussed in the main text for the IMI configuration performed with success. As a result, the corresponding current distribution and the radiation patterns are provided in Fig. 5.

For the sake of completeness, we used other plasmonic materials, such as silver and newly introduced TiN,[?] for the design of the patch antenna and the implementation of the cavity model in optics. The first example is a free-standing silver square patch ($a = b = 166$ nm and $d = 50$ nm) excited by a dipole ($\lambda_0 = 720$ nm) to operate in the TM_{10} mode ($x_0 = a/4, y_0 = b/2, z_0 = -15$ nm), for which the current distribution and the radiation patterns were obtained by the cavity model and the Maxwell solver, as given in Fig. 6.

The second example is a free-standing square TiN patch with the dimensions of $a = b = 145$ nm, $d = 25$ nm and excited by a dipole ($\lambda_0 = 620$ nm) to operate in the TM_{10} mode ($x_0 = a/4, y_0 = b/2, z_0 = -15$ nm), for which the current distribution and the radiation patterns were obtained by the cavity model and the Maxwell solver, as given in Fig. 7.

References

1. Gururaj V. Naik, Jeremy L. Schroeder, Xingjie Ni, Alexander V. Kildishev, Timothy D. Sands, and Alexandra Boltasseva, ”Titanium nitride as a plasmonic material for visible and near-infrared wavelengths,” Opt. Mater. Express 2, 478-489 (2012).

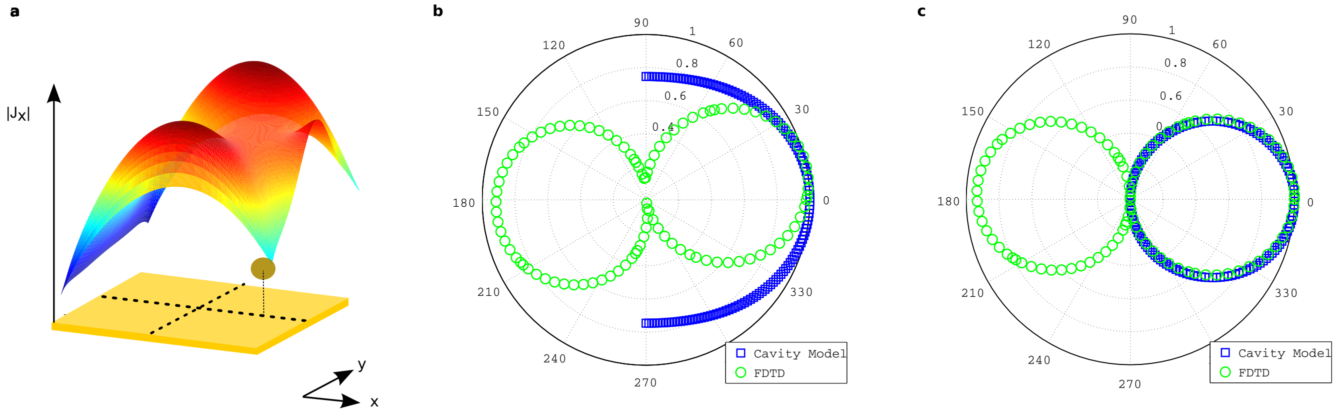

Figure 1. a) The current distribution, b) the radiation pattern in the E-plane and c) the radiation pattern in the H-plane for a square gold patch with $a = b = 270$ nm and $d = 50$ nm, when excited by a dipole ($\lambda_0 = 1100$ nm) for the TM_{10} mode at $(x_0 = 3a/4, y_0 = b/2, z_0 = -15$ nm).

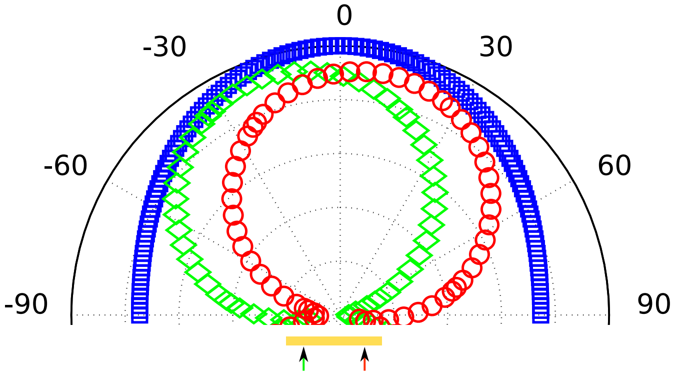

Figure 2. The radiation patterns in the E-plane for two symmetric locations of the feed (as shown in the inset) corresponding to the TM_{10} mode. The parameters of the geometry are as follows: $a = b = 270$ nm, $d = 50$ nm and $z_0 = -15$ nm. The data corresponding to the feed locations of $(x_0 = a/4, y_0 = b/2)$ and $(x_0 = 3a/4, y_0 = b/2)$ are represented by the green diamonds and red circles, respectively, whereas the blue squares represent the results obtained by the cavity model.

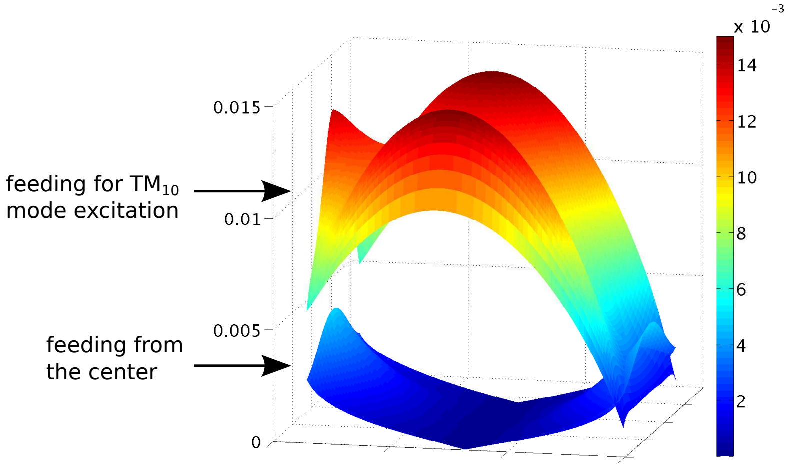

Figure 3. The magnitude of the current distributions for the patch fed by a dipole at $(x_0 = a/2, y_0 = b/2)$ and at $(x_0 = a/4, y_0 = b/2)$. The parameters of the geometry are as follows: $a = b = 270$ nm, $d = 50$ nm and $z_0 = -15$ nm.

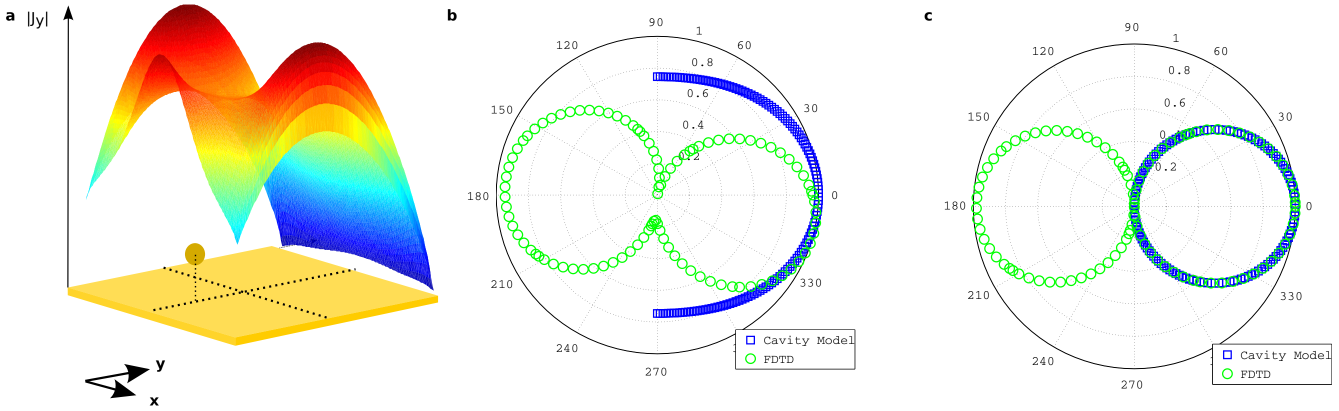

Figure 4. a) The current distribution, b) the radiation pattern in the E-plane and c) the radiation pattern in the H-plane for a square gold patch with $a = b = 270$ nm and $d = 50$ nm, when excited by a dipole ($\lambda_0 = 1100$ nm) for the TM_{01} mode at ($x_0 = a/2, y_0 = b/4, z_0 = -15$ nm).

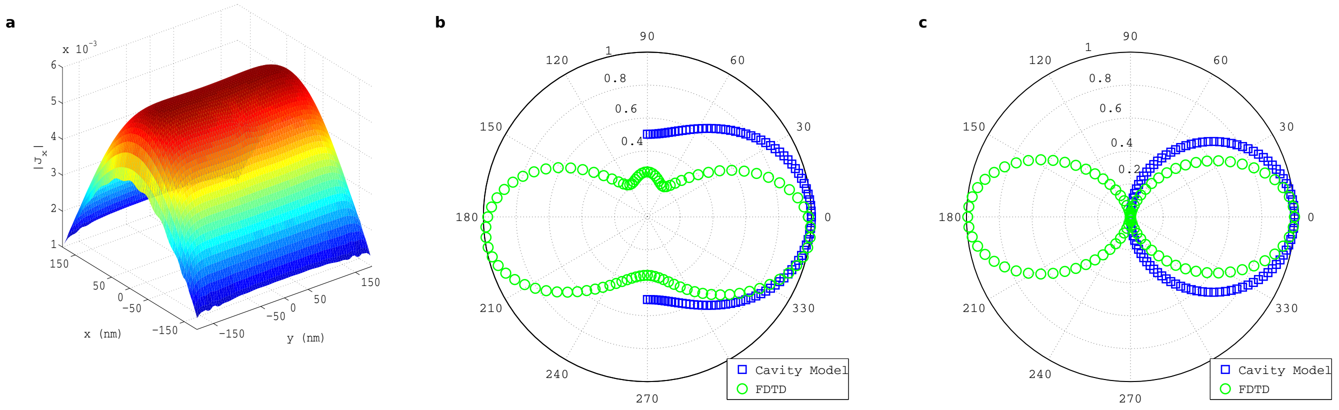

Figure 5. a) The current distribution, b) the radiation pattern in the E-plane and c) the radiation pattern in the H-plane for a square gold patch in an MIM configuration excited by a dipole ($\lambda_0 = 1100$ nm) for the TM_{10} mode operation. The parameters of the antenna are as follows: $a = b = 366$ nm, $d_{metal} = d_{air} = 50$ nm, ($x_0 = a/5, y_0 = b/2$) and $z_0 = -15$ nm defined as referenced to the bottom of the top metal patch.

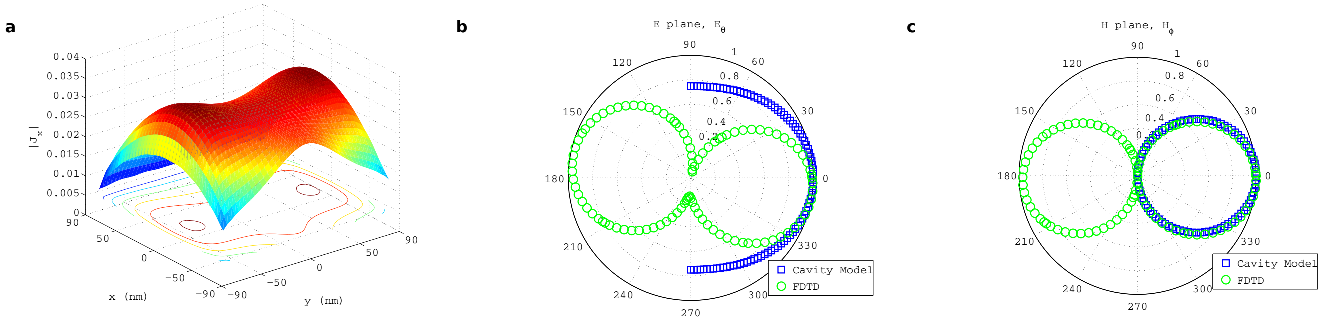

Figure 6. a) The current distribution, b) the radiation pattern in the E-plane and c) the radiation pattern in the H-plane for a square silver patch antenna with $a = b = 166$ nm and $d = 50$ nm, when excited by a dipole ($\lambda_0 = 1100$ nm) for the TM_{10} mode at ($x_0 = a/4, y_0 = b/2, z_0 = -15$ nm).

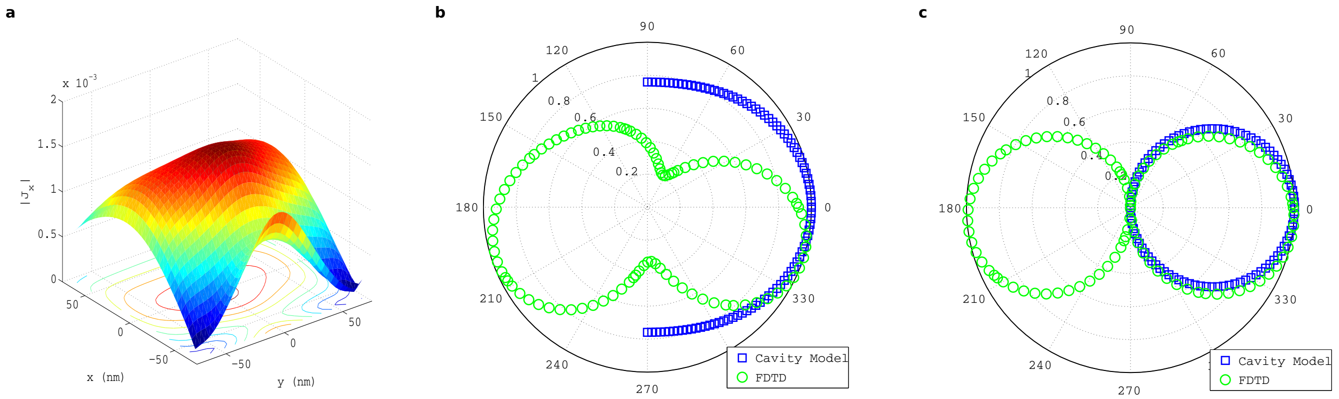

Figure 7. a) The current distribution, b) the radiation pattern in the E-plane and c) the radiation pattern in the H-plane for a square TiN patch antenna with $a = b = 145$ nm and $d = 25$ nm, when excited by a dipole ($\lambda_0 = 620$ nm) for the TM_{10} mode at ($x_0 = a/4, y_0 = b/2, z_0 = -15$ nm).
